# Plasma Ketone and Medium Chain Fatty Acid Response in Humans Consuming Different Medium Chain Triglycerides During a Metabolic Study Day

**DOI:** 10.3389/fnut.2019.00046

**Published:** 2019-04-16

**Authors:** Valérie St-Pierre, Camille Vandenberghe, Carolyne-Mary Lowry, Mélanie Fortier, Christian-Alexandre Castellano, Richard Wagner, Stephen C. Cunnane

**Affiliations:** ^1^Research Center on Aging, Sherbrooke, QC, Canada; ^2^Departments of Nuclear Medicine and Radiobiology, Université de Sherbrooke, Sherbrooke, QC, Canada; ^3^Departments of Medicine, Université de Sherbrooke, Sherbrooke, QC, Canada; ^4^Departments of Pharmacology and Physiology, Université de Sherbrooke, Sherbrooke, QC, Canada

**Keywords:** MCFA, ketones, human, acetoacetate, β-hydroxybutyrate, MCT

## Abstract

**Background:** Medium chain triglycerides (MCT) are ketogenic but the relationship between the change in plasma ketones and the change plasma medium chain fatty acids (MCFA)—octanoate, decanoate, or dodecanoate—after an oral dose of MCT is not well-known. An 8 h metabolic study day is a suitable model to assess the acute effects on plasma ketones and MCFA after a dose of tricaprylin (C8), tricaprin (C10), trilaurin (C12) or mixed MCT (C8C10).

**Objective:** To assess in healthy humans the relationship between the change in plasma ketones, and octanoate, decanoate and dodecanoate in plasma total lipids during an 8 h metabolic study day in which a first 20 ml dose of the homogenized test oil is taken with breakfast and a second 20 ml dose is taken 4 h later without an accompanying meal.

**Results:** The change in plasma acetoacetate, β-hydroxybutyrate and total ketones was highest after C8 (0.5 to 3 h post-dose) and was lower during tests in which octanoate was absent or was diluted by C10 in the test oil. The plasma ketone response was also about 2 fold higher without an accompanying meal (*P* = 0.012). However, except during the pure C10 test, the response of octanoate, decanoate or dodecanoate in plasma total lipids to the test oils was not affected by consuming an accompanying meal. Except with C12, the 4 h area-under-the-curve of plasma β-hydroxybutyrate/acetoacetate was 2–3 fold higher when no meal was consumed (*P* < 0.04).

**Conclusion:** C8 was about three times more ketogenic than C10 and about six times more ketogenic than C12 under these acute metabolic test conditions, an effect related to the post-dose increase in octanoate in plasma total lipids.

## Introduction

The ketones, β-hydroxybutyrate (β-HB) and acetoacetate (AcAc), are produced by the liver during fasting or dietary carbohydrate restriction. During long-term fasting, ketones can provide up to 80% of the brain's energy requirements (1–3). Aside from energy or carbohydrate restriction, one common way to increase plasma ketones is by ingesting medium chain triglycerides (MCT) that provide medium chain fatty acids (MCFA), i.e., saturated fatty acids of 6–12 carbons in chain length. Several different MCFA are present in mammalian milk and in coconut oil or palm oil (4–7). MCFA are ketogenic because they are more rapidly metabolized than long chain fatty acids. In contrast to long chain fatty acids (≥14 carbons), which are absorbed via the lymphatic system and incorporated into circulating chylomicrons before reaching the liver, 8 and 10 carbon MCFA are thought to reach the liver directly via the portal vein (3, 8–12). Eight carbon MCFA also cross the mitochondrial inner membrane without carnitine-dependent transport, allowing them to be more rapidly β-oxidized compared to long chain fatty acids (11).

Using a metabolic study day protocol, we have recently shown that tricaprin (C10 triglyceride) is significantly less ketogenic than tricaprylin (C8 triglyceride) (13). The changes in plasma MCFA under these study conditions have not yet been reported. The objectives of the present study were to assess: (i) changes in MCFA (octanoate, decanoate and dodecanoate) in plasma total lipids after various MCT treatments during a metabolic study day, (ii) how changes in octanoate, decanoate, and dodecanoate in plasma total lipids were related to changes in plasma ketones and the plasma ratio of BHB/AcAc during the metabolic study day, and (iii) the ketogenic effect of trilaurin (C12 triglyceride). The plasma MCFA analyses were all done on plasma total lipids.

Previous studies with ketogenic therapies, (diet, supplements or iv infusion), showed beneficial effects on cognitive outcomes (14–18). A better understanding of the plasma MCFA and ketone response to various MCT could potentially help improve the formulation of ketogenic MCT supplements and might also provide a way of evaluating the compliance of participants taking such supplements in clinical trials, especially in an older population experiencing cognitive decline.

## Methods

### Participants

Ethical approval for this study was obtained from the Research Ethics Committee of the Integrated University Health and Social Services of Eastern Townships—Sherbrooke University Hospital Center, which oversees all human research done at the Research Center on Aging (Sherbrooke, QC, Canada). All participants provided written informed consent prior to starting the study and were recruited from August 2015 to October 2017. They underwent a screening visit, including the analysis of a blood sample collected after a 12 h overnight fast. Exclusion criteria included smoking, diabetes or glucose intolerance (fasting glucose > 6.1 mM and glycosylated hemoglobin > 6.0%), strenuous aerobic exercise more than three times a week, coconut oil allergy, untreated hypertension, dyslipidemia, or abnormal renal, liver, heart or thyroid function. This project is registered on ClinicalTrials.gov (NCT 02679222).

### Test Oils

For clarity, the abbreviations—C8, C10, and C12—will be used when referring to the dietary supplement provided, whereas the full names—octanoate, decanoate, and dodecanoate—will be used when referring to the plasma levels of these MCFA. The test oils were provided by Abitec Corporation, Columbus, OH, USA (Table 1); the C8 oil was 91% pure tricaprylin (Captex 8000), the C10 oil was 91% pure tricaprin (Captex 1000), the C12 oil was 95% pure trilaurin, and the MCT oil was a mixture of 55% C8 and 35% C10 (Captex 355; referred to here as “C8C10.” A 20 mL dose of each test oil was homogenized into 250 mL of lactose-free skim milk (Natrel®, Longueuil, QC, CAN) using a kitchen blender (Magic Bullet®, Los Angeles, CA, USA) in order to compare with our previously published studies (19). C10 and C12 are solid at room temperature so they were melted in a water bath at 60°C prior to being homogenized into the lactose-free skim milk vehicle.

**Table 1:** Volume of the test oils given on the metabolic study days.

	**Quantity (ml)**			
	**C8**	**C10**	**C12**	**Other**
CTL	0	0	0	0
C8	18.2	0	1	0.8
C8C10	10	7	1.8	1.2
C10	0	18.2	0.9	0.9
C12	0	0	19	1

*Each test dose provided a total of 20 ml of the test substance. CTL, control; C8, tricaprylin; C10, tricaprin; C12, trilaurin; C8C10 (55% C8 and 35% C10)*.

### Experimental Design

The protocol involved five separate metabolic study days for each participant, so was a repeated, longitudinal measurements design during which the test substances were evaluated in random order: vehicle (CTL; 250 mL lactose-free skim milk; 80 calories/drink) or 20 mL of the test substance mixed with 250 mL of lactose-free skim milk (C10, C8, C8C10, or C12; total of 220 calories/drink). Participants were blinded to the treatments on test days and crossed over from one treatment to the next with a minimum 3 day wash-out between tests. The test sequence was determined *a priori* by the investigator. On each metabolic study day, participants arrived at 7:30 a.m. after a 12 h overnight fast and a minimum of 24 h without alcohol intake. A forearm venous catheter was inserted and a fasting baseline blood sample was withdrawn (Time 0). At about 8 a.m., participants received the standard breakfast during which they also consumed the test or CTL drink. The breakfast consisted of two pieces of toast with raspberry jam, a piece of cheese, and two scrambled eggs (total of 470 calories, 19.5 g of fat, 24.2 g of protein and 55 g of carbohydrate). Four hours later (at about noon), a second dose of the test drink was given but with no other food (Table 1). Water was available *ad libitum* throughout the study day. Venous forearm blood samples were taken in EDTA tubes as baseline (pre-dose) and every 30 min during the 8 h study period, with the first post-dose sample taken 30 min after the test drink was consumed. Blood samples were centrifuged at 2846 G for 10 min at 4°C and plasma stored at −80°C until analyzed.

### Plasma Metabolite Analyses

Plasma β-HB and AcAc were measured by an automated colorimetric assay as previously described (6). Briefly, for AcAc, 25 μL of plasma was mixed with 330 μL of fresh reagent (Tris buffer, pH 7.0, 100 mM, 20 mM sodium oxamate; 0.15 mM NADH and 1 U/mL β-hydroxybutyrate dehydrogenase [β-HBDH]). For β-HB, the reagent was Tris buffer (pH 9.0; 20 mM sodium oxamate, 1 mM NAD, and 1 U/mL β-HBDH). Tris, oxamic acid, DL-β-HB sodium salt, Li-AcAc standard, and NAD were purchased from Sigma (St. Louis, MO, USA), NADH, from Roche (Mannheim, Germany), and β-HBDH from Toyobo (Osaka, Japan). The change in absorbance at 340 nm between 15 and 120 s after the addition of the reagent was measured on an automated clinical chemistry analyzer (Dimension Xpand Plus; Siemens, Deerfield, IL, USA). The assay was calibrated with freshly diluted standards from frozen aliquots of a 10 mM standard of Li-AcAc or DL-β-HB sodium salt, which is stable at −20°C for 2 and 6 months, respectively. Calibrations and quality controls were performed for each assay to ensure the precision of the assays (coefficient of variation between tests 5 ± 1%). Where plasma “total ketones” are reported, this refers to the total of AcAc and β-HB combined.

### Measurement of Plasma Octanoate, Decanoate, and Dodecanoate

Samples for analysis of octanoate, decanoate, and dodecanoate in plasma total lipids were prepared with modification of a previously reported method (20, 21). Plasma samples (25 μL) were spiked with 10 μL of the isotopic standards, 1, 2, 3, 4-^13^C_4_ octanoic acid, methyl-D_3_ decanoic acid and methyl-D_3_ dodecanoic acid. After mixing 5 μL of 9 M KOH with a 25 μL sample of plasma, the tubes were place in a water bath at 60°C for 0.5 h. After adjusting the pH by adding 20 μL of 2.25 M HCl, 450 μL of acetonitrile was added and the samples centrifuged at 16,400 g for 0.5 h. Finally, 80 μL of the supernatant was added to 120 μL of 5 mM ammonium bicarbonate and the sample stored at 4°C until analysis. MCFA analysis was performed by ultra-high performance liquid chromatography (Nexera X2, Shimadzu) coupled to a tandem mass spectrometer (API-3000, ABSciex). The chromatography was carried out using an Acquity UPLC HSS T3 1.8 μm column fitted with a BEH C18 1.7 μm VanGuard pre-column both maintained in a heating compartment at 30°C. The MCFA were eluted by a binary gradient starting at 75% solvent A and 25% solvent B, and increasing linearly to 100% solvent B for 5 min, at a flow rate of 0.5 mL/min. Solvent A was an aqueous solution of ammonium bicarbonate (5 mM) adjusted to pH 6 by the addition of formic acid, and solvent B was 90% acetonitrile in water. The gradient was held at 100% solvent B for 4 min before re-equilibrating for another 4 min under the initial conditions. A 10 μL injection volume was employed. Under these conditions, octanoate, decanoate, and dodecanoate eluted at approximately 2.5, 3.3, and 3.6 min, respectively. The natural and isotopic derivatives of the MCFA were detected by tandem MS in negative mode using MRM with both quadruples (Q1 and Q3) set to the corresponding molecular ions: natural octanoate (m/z 143); isotopic octanoate (m/z 147); natural decanoate (m/z 171); isotopic decanoate (m/z 174); natural dodecanoate (m/z 199); isotopic dodecanoate (m/z 203). The source temperature was 500°C and the collision energy was set to −15 on the instrument. The MCFA concentration in the plasma samples was determined from the ratio of the natural compound in question to isotopic standard added prior to sample preparation. Under these conditions, the lower detection limit for each of plasma octanoate, decanoate and dodecanoate was ≤1.85 μmol/L.

### Statistical Analysis

All results are given as the mean ± SEM. The sample size calculation was based on a previous study in which 8 participants were sufficient to measure a significant difference (β = 0.80) in plasma ketones after consuming 30 g of MCT (6). We recruited *n* = 9 for the present study in case of a dropout during one of the tests. All statistical analyses were carried out using SPSS 23.0 software (SPSS Inc., Chicago, IL, USA). Ketone data obtained after the CTL test were subtracted from the ketone data obtained after the various test substances. The areas-under-the-curve (AUC) for plasma total ketones, octanoate, decanoate, and dodecanoate were calculated according the trapezoid method (22). The AUCs were separated into 0–4 h and 4–8 h periods because there was a meal given with the test substance at time 0 but not at 4 h. Since the data were not normally distributed (*n* < 30/point), results of the tests were compared using Friedman's test, and the effect of the treatments as well as changes over time for each treatment were determined using Wilcoxon's signed rank test. Spearman correlations were used to measure the statistical dependence between two variables. Differences were considered statistically significant at *P* ≤ 0.05. Graphs were prepared using Prism version 6.0 (GraphPad Software Inc., San Diego, CA, USA).

## Results

A total of *n* = 9 people (*n* = 7 men and *n* = 2 women) completed all the tests except for C10 (*n* = 8; Table 2). The participants' baseline biochemical parameters corresponded to normal reference values from the Sherbrooke University Hospital Center (Sherbrooke, QC).

**Table 2:** Baseline demographic and biochemical parameters of the participants.

**CHARACTERISTICS**
Age (y)	34 ± 12^a^
Weight (kg)	72 ± 10
Height (cm)	175 ± 6
Body mass index (kg/m^2^)	24 ± 3
***Plasma metabolites***	
Glucose (mmol/L)	4.3 ± 0.2
Total ketones^b^ (μmol/L)	90 ± 61
Glycated hemoglobin (%)	5.2 ± 0.4
Total cholesterol (mmol/L)	4.3 ± 0.9
Triacylglycerols (mmol/L)	0.7 ± 0.3

a *Values are mean ± SEM, n = 9*.

b *Acetoacetate and β-hydroxybutyrate combined*.

### MCFA in Plasma Total Lipids

**Figure 1:**
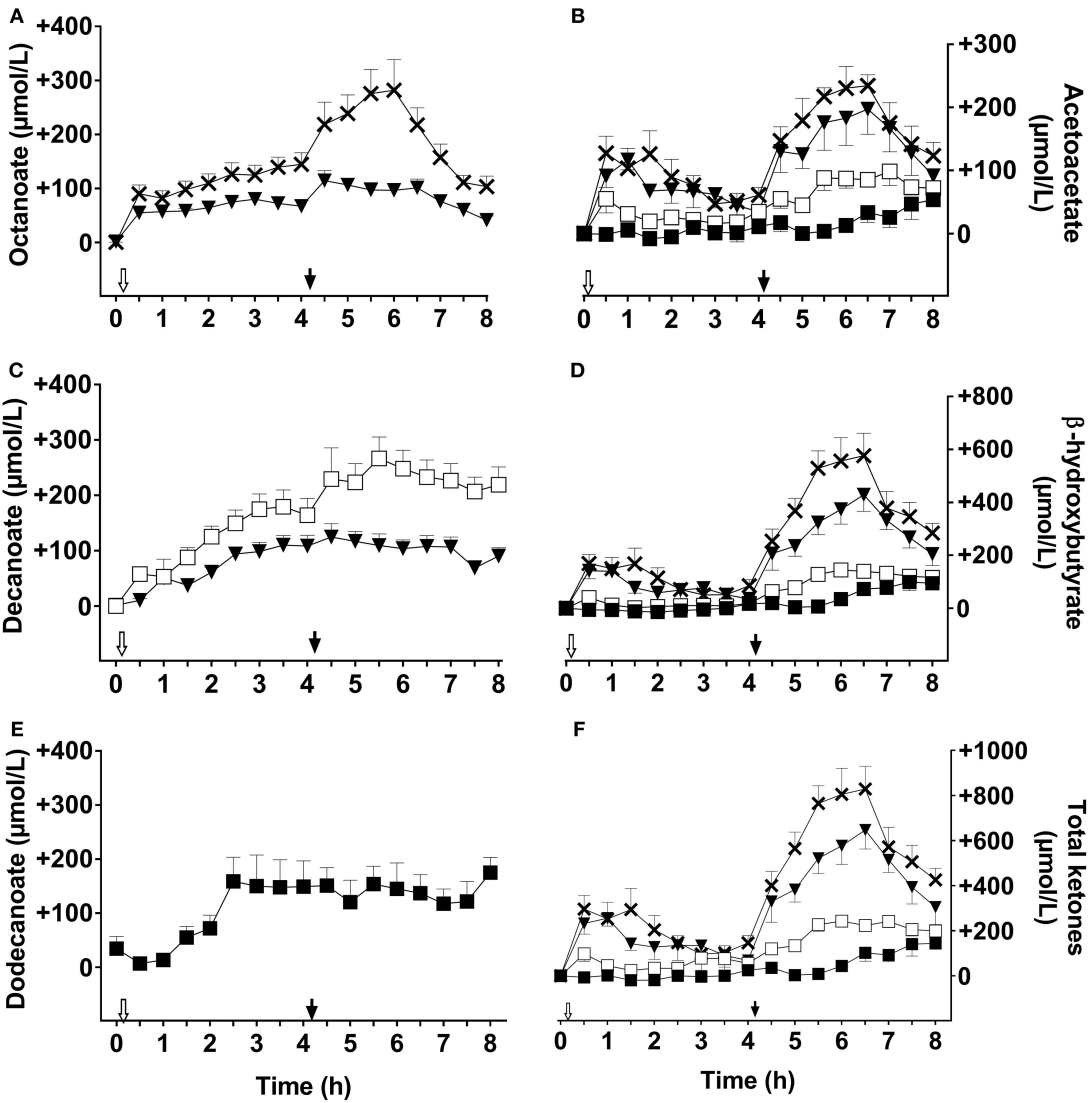
Plasma octanoate **(A)**, acetoacetate **(B)**, decanoate **(C)**, β-hydroxybutyrate **(D)**, dodecanoate **(E)**, and total ketones **(F)** throughout the 8 h metabolic study day during which two 20 ml doses of the following test substances were consumed: tricaprylin (C8,×), tricaprin(C10,□), medium chain triglyceride (C8C10, ▾), or trilaurin (C12, ■). Plasma acetoacetate, β-hydroxybutyrate, and total ketones values were normalized to T0, and control day values were subtracted prior to graphing the results. Plasma octanoate, decanoate, and dodecanoate were extracted from plasma total lipids. The white arrow (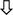) indicates the time at which breakfast plus the dose of test oil was consumed; the black arrow (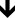) indicates when the dose of test oil alone was consumed at midday (without a meal). Data are the mean ± SEM for *n* = 8–9 participants/point.

Plasma octanoate and decanoate were not detectable during the CTL test (data not shown). The following data are reported as the difference above CTL values. The breakfast dose of C8 increased plasma octanoate by 90 ± 35 μmol/L within 30 min of ingestion and the values increased to a maximum of 145 ± 53 μmol/L above CTL after 4 h (Figure 1A). The second dose of C8 given 4 h later further increased plasma octanoate for another 2 h, peaking at 282 ± 119 μmol/L. Averaged over the whole C8 test day, mean plasma octanoate was 148 ± 41 μmol/L about 2 h after the second dose. C8C10 also increased plasma octanoate over the metabolic study day, reaching 80 ± 29 μM 3 h after the first dose in the morning, and 115 ± 40 μmol/L 30 min after the second dose. The overall day-long mean of plasma octanoate was 2 fold higher on C8 compared to C8C10. The C10 had no significant effect on plasma octanoate (data not shown).

C10 increased plasma decanoate throughout the metabolic study day, peaking at 267 ± 79 μmol/L about 1 h after the second dose of C10 (Figure 1C). C8C10 also increased plasma decanoate but to a maximum of 125 ± 57 μmol/L 30 min after the second dose, which was about half of that attained with C10. Plasma decanoate was not significantly influenced by consuming C8 (data not shown). After the initial dose of C12, plasma dodecanoate increased to 160 ± 93 μmol/L where it stayed without changing after the second dose of C12. C12 did not significantly influence plasma octanoate or decanoate (data not shown).

### Area-Under-the-Curve for MCFA and Ketones

**Figure 2:**
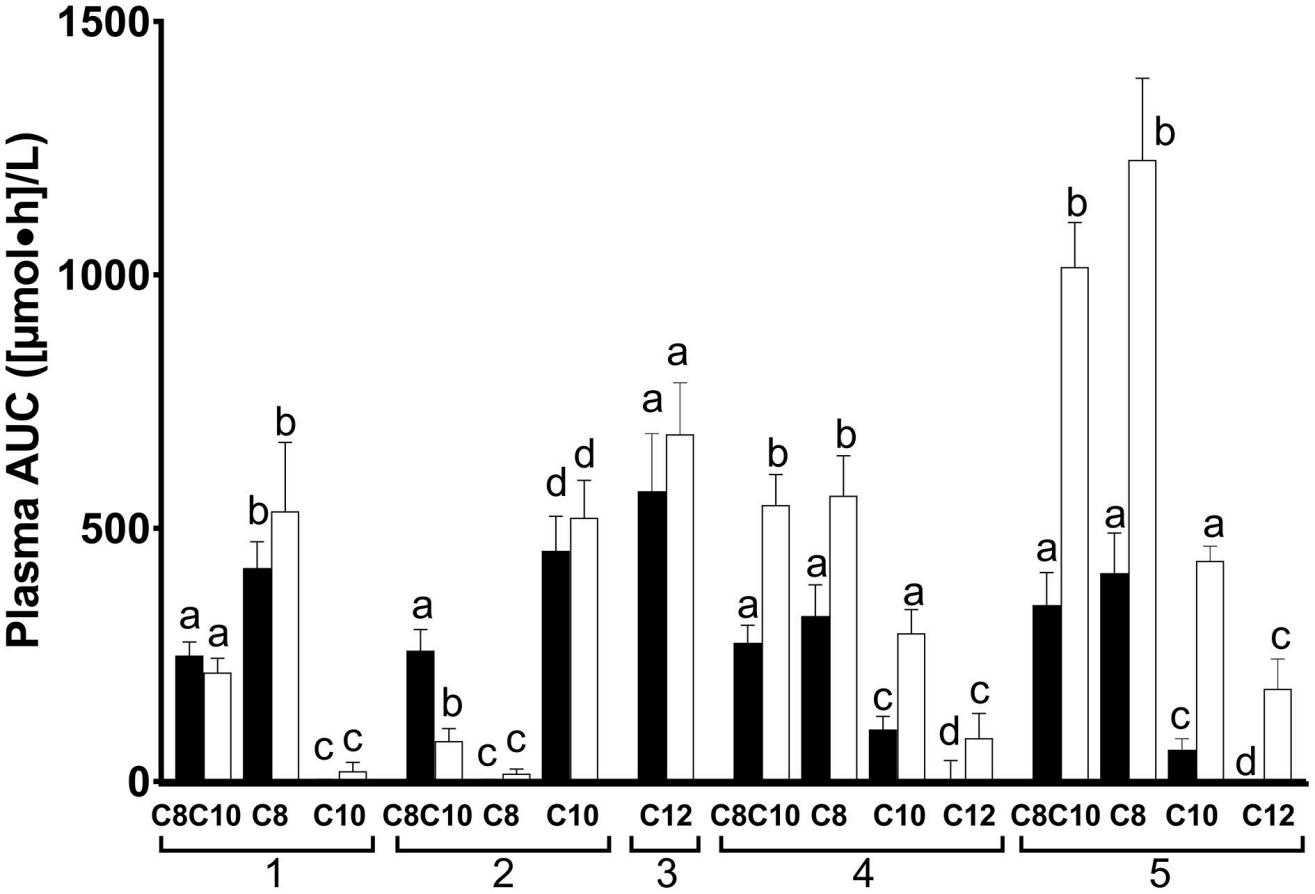
Area-under-the-curve (AUC) of plasma octanoate (1), decanoate (2), dodecanoate (3), acetoacetate (4), and β-hydroxybutyrate (5) during the metabolic study day on which the doses of medium chain triglyceride (C8C10), tricaprylin (C8), tricaprin (C10), or trilaurin (C12) were tested. Plasma MCFA were extracted from total plasma lipids. AUC data are shown in pairs of bars: the left-hand bar of each pair represents the data for the 0–4 h AUC while the right-hand bar represents the 4–8 h AUC. Bars are the mean ± SEM for *n* = 8–9. For each metabolite shown, the 0–4 h AUC was significantly different from the 4–8 h AUC where letters are different (*P* < 0.05).

The AUCs for plasma ketones, octanoate, decanoate and dodecanoate differed significantly according to the test substance given (*P Friedman* ≤ 0.002, Figure 2). As would be expected by the higher content of octanoate in C8, the 0–4 h AUC of plasma octanoate was about twice as high for C8 as for C8C10 (*P* = 0.004, Figure 2) and 104 fold higher after C8 than after C10 (*P* = 0.006). Again, as would be expected, the 0–4 h AUC for plasma decanoate was twice as high after C10 as it was for C8C10 (*P* = 0.009, Figure 2), and was 189 fold higher than after C8 (*P* = 0.006). The 4–8h AUC for plasma octanoate was 1.5 fold and 24 fold higher for C8 compared to C8C10 or to C10, respectively (*P* ≤ *0.01*). C10 increased the 4–8 h plasma decanoate AUC by 5.5 fold and 32 fold when compared to C8C10 or C8, respectively (*P* = *0.006*). The AUC for plasma dodecanoate did not differ significantly from 0–4 h to 4–8 h.

AUCs for plasma total ketones were not significantly different between C8 and C8C10. Compared to C10 given alone, both C8 and C8C10 increased the AUCs for plasma AcAc by 2 fold, plasma β-HB by 5 fold and total ketones by 2.5 fold (*P* ≤ 0.028) for the first half of the metabolic study day, and by 1, 1.5, and 2 fold during the second half of the study day, respectively (*P* = 0.012). Compared to C10, AUCs for plasma AcAc and β-HB after C12 were 20 and 62 fold lower, respectively, (*P* ≤ 0.018) during the 0–4 h period and were 2.5 and 1.5 fold lower, respectively, during the 4–8 h period (*P* ≤ 0.009).

There was a >6 fold difference across treatments in the ratio of the plasma AUC for β-HB/AcAc during the 0–4 h period, with C12 having the highest ratio, CTL equal to C10 with the lowest ratio, and C8 and C8C10 in between (Table 3). The ratio of the plasma AUC for β-HB/AcAc was uniformly 2–3 fold higher across treatments during the 4–8 h period (no meal) compared to the 0–4 h period (with meal) with the exception of C12 for which this ratio was 20% lower compared to the 0–4 h period (*P* < *0.04*).

**Table 3:** Area-under-the-curve (AUC) of the ratio of plasma β-hydroxybutyrate/acetoacetate during the first half (0–4 h; with breakfast) and second half (4–8 h; no lunch) of the metabolic study day^1^.

	**0–4 h**	**4–8h**
CTL	0.53 ± 0.15^acde^	1.93 ± 0.24^adf^
C8^*^	1.27 ± 0.07^b^	2.42 ± 0.30^ace^
C8C10^*^	1.20 ± 0.13^b^	1.98 ± 0.13^adg^
C10^*^	0.60 ± 0.12^ad^	1.41 ± 0.12^bce^
C12	3.37 ± 3.27^abcde^	2.70 ± 5.9^bfg^

1 *Values are mean ± SEM, n = 8–9/data point*.

*See legend to Table 1 for abbreviations*.

*Letters in the same column are different when the values were significantly different from one supplement to another (P ≤ 0.05)*.

* *All conditions were different between AUC 0–4 h vs. 4–8 h (P ≤ 0.043) except for CTL and C12 (P > 0.106)*.

### Correlation Between Plasma Total Ketones and Individual Plasma MCFA

**Figure 3:**
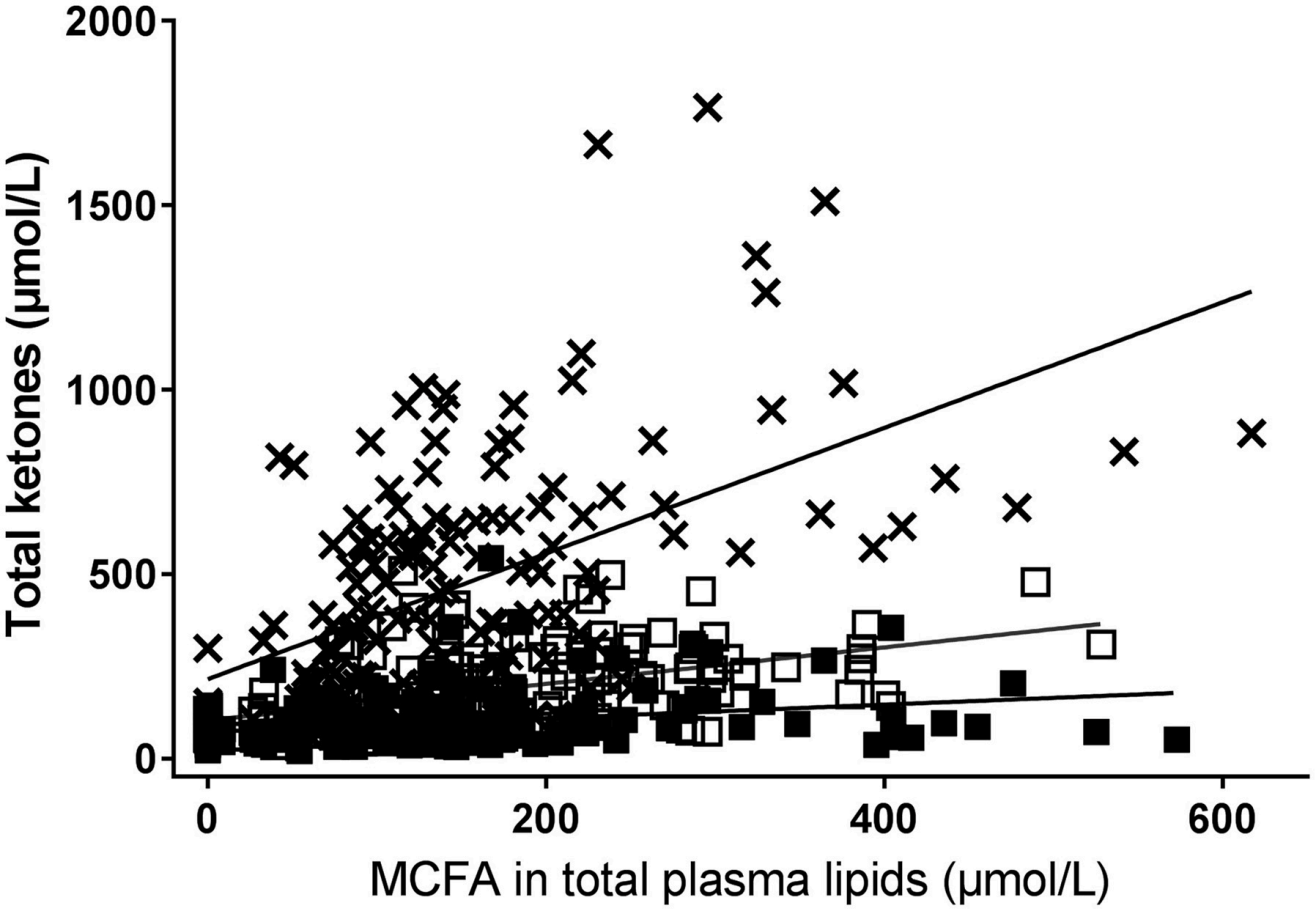
Correlation between plasma MCFAs (octanoate [×], decanoate [□], or dodecanoate [■]) and plasma total ketones after consuming a dose of the following test substances with breakfast or the test substance alone at midday during a 8 h metabolic study day: tricaprin (C10, □ ρ = 0.56, *P* < 0.0001), tricaprylin (C8, × [A] ρ = 0.57, *P* < 0.0001) or trilaurin (C12, ■ [C] ρ = 0.37, *P* = < 0.0001). Plasma MCFA were extracted from total plasma lipids. Total plasma ketones = acetoacetate + β-hydroxybutyrate.

The increase in plasma total ketones was significantly positively correlated to the increase in MCFA in plasma total lipids regardless of the test substance given (Figure 3). However, the correlation with the change in plasma ketones was strongest after C8 (ρ = 0.57, *P* < 0.0001) than after C10 (ρ = 0.35, *P* < 0.0001) or C12 (ρ = 0.37, *P* ≤ 0.0001). The slope of the correlation of plasma ketones to MCFA in plasma total lipids was about three times higher after C8 than after C10 and about nine times higher after C8 than after C12 (Figure 3).

## Discussion

This study demonstrates that the significantly greater ketogenic effect in humans of an oral dose of C8 compared to C10 (13) or C12 is due to a difference in their effect on ketogenesis, and not to substantially different increases in the levels of octanoate, decanoate or dodecanoate in plasma total lipids after an oral dose of these MCT. We also show here that, in relation to changes in plasma MCFA, C8 has a significantly stronger dose response effect on plasma ketones than C10 or C12. For example, at 400 μM of each MCFA in plasma total lipids, plasma ketones were about three times higher after C8 than after C10 and two times higher after C10 than C12 (Figure 3). Indeed, C10 and C12 were not statistically significantly more ketogenic than the CTL condition. Plasma octanoate and decanoate were almost undetectable in all our baseline or CTL test samples so the presence of these two MCFA in plasma total lipids appears to depend on them being consumed quite recently.

The design of our metabolic study day protocol permitted us to evaluate the effect of consuming a meal on the plasma ketone and MCFA responses to the test substances. Compared to not consuming a meal at midday, consuming a breakfast containing 470 kilocalories with the 20 ml dose of C8, C10, or C12 did not significantly affect the AUC of plasma octanoate, decanoate or dodecanoate but did significantly reduce the AcAc and β-HB AUCs by ≥50% for the tests with C8C10, C8, C10, or C12 compared to CTL (Figure 2). Hence, a carbohydrate-rich test breakfast does not appear to affect absorption of octanoate, decanoate, or dodecanoate or their level in plasma total lipids. However, the breakfast did reduce the conversion of these MCFA to ketones during the following 3–4 h, possibly due to post-prandial insulin secretion. This may have implications for planning long-term ketogenic interventions based on MCT because, in order to maximize the resulting ketosis, it may be advantageous to give such interventions without a meal, i.e., as a stand-alone evening snack or as a replacement for breakfast. On the other hand, the meal may be beneficial because it tends to dampen the transitory gastrointestinal discomfort commonly seen with MCT, an effect that was not formally evaluated in this study. Plasma ketones returned toward baseline relatively quickly after C8 or C8C10 but plasma octanoate, decanoate and dodecanoate remained elevated for at least 8 h (Figure 1), suggesting that the spot checks of plasma MCFA could have some utility for monitoring compliance when one or other of these fatty acids is consumed in a longer term study.

The differential effect of the various test oils on the AUC of the ratio of β-HB/AcAc in plasma is novel and intriguing (Table 3). Not consuming a meal (4–8 h in the metabolic study day) roughly doubled the β-HB/AcAc ratio regardless of the test oil. Despite a very weak net ketogenic effect, C12 increased this ratio the most such that it was nearly 6 times higher than CTL taken at breakfast. The biological implications of these differences across our treatments are unclear. Plasma β-HB and AcAc are in an equilibrium controlled principally by βHBDH so conditions favoring higher AcAc must drive the reaction from β-HB toward AcAc. Generally, a higher redox potential at the cellular level is associated with higher ATP and better energy status (23, 24). However, it is difficult to extrapolate from a plasma ratio of β-HB/AcAc to mitochondrial redox status in a specific organ or cell so the biological significance of 5–6 fold differences in this ratio and the effect of the meal observed under the present conditions is unclear. Ketogenic interventions have clinically important uses in conditions such as intractable epilepsy but their clinical benefit is not necessarily directly associated with the overall level of ketosis (25). Whether some clinical outcome could be associated with higher plasma total ketones but lower plasma β-HB/AcAc (or the reverse) therefore awaits further study.

One limitation of this study is the single day metabolic study design because it is unknown whether these results can be extrapolated to longer term consumption of MCT. A further limitation is that the plasma MCFA were analyzed from plasma total lipids, so the extent to which the MCFA were in the free fatty acid vs. esterified form is not known. It may be that the better correlation between plasma octanoate and ketones than between plasma decanoate or dodecanoate and plasma ketones is due to more plasma octanoate remaining as the free fatty acid (26).

In conclusion, C8 was the most ketogenic supplement evaluated in these one day metabolic tests. C10 was poorly ketogenic but may promote glycolysis and lactate release in astrocytes, which could improve mitochondrial function and contribute to an anti-epileptic effect by inhibiting the AMPA receptor (27–29). Further research is needed to determine whether there are biological effects of C8, C10, or C12 in humans independent of their effects on ketogenesis.

## Ethics Statement

This study was carried out in accordance with the recommendations of Research Ethics Committee of the Integrated University Health and Social Services of Eastern Townships–Sherbrooke University Hospital Center, with written informed consent from all subjects. All subjects gave written informed consent in accordance with the Declaration of Helsinki. The protocol was approved by the Research Ethics Committee of the Integrated University Health and Social Services of Eastern Townships—Sherbrooke University Hospital Center.

## Author Contributions

SC, C-AC and MF: designed the study. CV and VS-P: conducted the study. CV, VS-P, C-ML, RW and SC analyzed and interpreted the data. VS-P and SC drafted and revised the manuscript. All the authors read and approved the final version of the paper.

### Conflict of Interest Statement

SC has consulted for and/or received travel support from Pruvit, Nisshin Oillio and Accera, has received test materials for research from Nestlé, Abitec, Bulletproof and HVMN, and has received funding for research from Nestlé. SC is president and sole shareholder of Senotec, a company developing keto-neurotherapeutics. The remaining authors declare that the research was conducted in the absence of any commercial or financial relationships that could be construed as a potential conflict of interest.

## Abbreviations

β-HB: β-hydroxybutyrate
AcAc: acetoacetate
MCT: medium chain triglyceride
C8: tricaprylin
C10: tricaprin
C12: trilaurin
MCFA: medium chain fatty acid.
